# Water limitation intensity shifts carbon allocation dynamics in Scots pine mesocosms

**DOI:** 10.1007/s11104-023-06093-5

**Published:** 2023-06-17

**Authors:** Emily F. Solly, Astrid C. H. Jaeger, Matti Barthel, Roland A. Werner, Alois Zürcher, Frank Hagedorn, Johan Six, Martin Hartmann

**Affiliations:** 1 Department of Environmental Systems Science, Sustainable Agroecosystems Group, ETH Zürich, Universitätstrasse 2, 8092 Zurich, Switzerland; 2 Department of Environmental Systems Science, Grassland Sciences Group, ETH Zurich, Universitätstrasse 2, 8092 Zurich, Switzerland; 3 Swiss Federal Institute for Forest, Snow and Landscape Research WSL, Biogeochemistry Group, Zürcherstrasse 111, Birmensdorf, 8903 Switzerland

**Keywords:** ^13^C Pulse Labelling, Carbon Allocation Belowground, Mesocosms, *Pinus sylvestris*, Plant Growth, Soil Water Limitation

## Abstract

**Background and aims:**

Tree species worldwide suffer from extended periods of water limitation. These conditions not only affect the growth and vitality of trees but also feed back on the cycling of carbon (C) at the plant-soil interface. However, the impact of progressing water loss from soils on the transfer of assimilated C belowground remains unresolved.

**Methods:**

Using mesocosms, we assessed how increasing levels of water deficit affect the growth of *Pinus sylvestris* saplings and performed a ^13^C-CO_2_ pulse labelling experiment to trace the pathway of assimilated C into needles, fine roots, soil pore CO_2,_ and phospholipid fatty acids of soil microbial groups.

**Results:**

With increasing water limitation, trees partitioned more biomass belowground at the expense of aboveground growth. Moderate levels of water limitation barely affected the uptake of ^13^C label and the transit time of C from needles to the soil pore CO_2_. Comparatively, more severe water limitation increased the fraction of ^13^C label that trees allocated to fine roots and soil fungi while a lower fraction of ^13^CO_2_ was readily respired from the soil.

**Conclusions:**

When soil water becomes largely unavailable, C cycling within trees becomes slower, and a fraction of C allocated belowground may accumulate in fine roots or be transferred to the soil and associated microorganisms without being metabolically used.

**Supplementary Information:**

The online version contains supplementary material available at 10.1007/s11104-023-06093-5.

## Introduction

Trees are typically adapted to cope with multiple climatic disturbances such as water limitation, fires, and windthrow. Yet worldwide, trees are facing a human-related intensification of these stresses (Millar and Stephenson 2015; Trumbore et al. 2015). In particular, episodes of water limitation are becoming more frequent and severe and can affect tree functioning through alterations in the allocation of photosynthetically fixed carbon (C) to different aboveground and belowground components (IPCC 2021; Joseph et al. 2020; McDowell et al. 2020; Weemstra et al. 2013). Trees are generally predicted to allocate more C to root tissues with reductions in soil water availability (Bloom et al. 1985; Ledo et al. 2018; Poorter et al. 2012). However, such a response may depend on the severity of the limitation (Hartmann et al. 2020, 2013; Ruehr et al. 2009). Furthermore, although a portion of the C assimilated through photosynthesis is commonly deposited by roots into the soil (Brunn et al. 2022; Pausch and Kuzyakov 2018; Rog et al. 2021), little is known about whether the intensity of soil water deficit affects the transfer of C from plants to soils and associated soil microorganisms (Prescott et al. 2020).

Tree growth is often constrained by environmental conditions that lead to low availability of soil water (McDowell et al. 2020; Weemstra et al. 2013). Evidence shows that in response to reductions in soil water availability, aboveground plant growth and respiration usually tend to decrease at an earlier stage of limitation than photosynthesis (Hsiao et al. 1976; Muller et al. 2011; Palacio et al. 2014). Trees would thereby produce more photosynthetic assimilates (source activity) than needed to support their metabolic functions (sink activity) (Prescott et al. 2020), which may in turn feed back on photosynthesis due to a reduced C demand (Gessler and Grossiord 2019; Hagedorn et al. 2016). In leaf tissues, some of the assimilated C is converted to metabolites and carbohydrates involved in osmoregulation or storage (Hartmann and Trumbore 2016). These compounds are transferred through the phloem from leaves to other tissues, including root systems, and a portion of C allocated to roots can be deposited into the soil as rhizodeposits. Rhizodeposits include root-released cells, exudates, and lysates known to fuel the metabolism of soil microorganisms (Dennis et al. 2010; Tian et al. 2020).

Recent research indicates that the velocity at which C metabolites and carbohydrates are transported belowground is reduced in trees and perennial herbaceous plants that are limited by water (Gao et al. 2021; Ingrisch et al. 2020; Salmon et al. 2019). This reduction is assumed to be mainly related to a delay in the export of C from leaves as well as an increased sap viscosity and a decrease in phloem turgor (Dannoura et al. 2019; Ruehr et al. 2009; Salmon et al. 2019; Sevanto 2014; Sevanto et al. 2014). Nevertheless, a reduced metabolic activity in roots under unfavorable soil moisture levels may lead to a build-up of storage carbohydrates and metabolites in belowground plant tissues (Hagedorn et al. 2016; Oberhuber et al. 2011). Mean residence times of C estimated from radiocarbon (^14^C) measurements provide consistent evidence that in woody species C can be stored for multiple years and used at a later point in time for respiratory metabolism and growth (Hartmann and Trumbore 2016; Herrera-Ramírez et al. 2020; Hilman et al. 2021; Muhr et al. 2013; Richardson et al. 2015; Solly et al. 2018). However, knowledge is currently lacking on how water limitation affects the transit time and accumulation of C in trees and its cascading effects on belowground plant growth and rhizodeposition (Solly et al. 2018).

Tree species adapted to dry conditions tend to sustain longer-lasting root organs to optimize water uptake (Brunner et al. 2015; Herzog et al. 2014). Nevertheless, relative changes in the partitioning between below- and aboveground biomass likely depend on the severity of the water stress and the growth of other plant tissues. Trees exposed to severe water limitation generally reduce their aboveground growth (Poorter et al. 2012). This decrease in aboveground growth can, in turn, lead to an increase in the fraction of belowground biomass relative to the total biomass of trees. Under moderate soil water deficit, trees have instead been observed to maintain their aboveground growth for as long as possible with only minor alterations in root growth (Poorter et al. 2012). However, divergent responses to water depletion have been observed for roots of diverse diameter sizes within the same root system of trees (Brunner et al. 2015; Olmo et al. 2014).

The most dynamic responses of plant root systems are expected for the most ephemeral roots with a narrow diameter (here defined as fine roots, < 2 mm in diameter) (Iversen et al. 2017; Jackson et al. 1990; Matamala and Stover 2013; Solly et al. 2013; Trumbore and Gaudinski 2003). This is because fine roots are responsible for the acquisition of water and nutrients from the soil*.* The growth and morphology of fine roots do not only depend on genetically determined species characteristics but also on the distribution of resources in the soil matrix (Comas et al. 2012; Imada et al. 2008; Iversen 2010; Jobbágy and Jackson 2000; Malhotra et al. 2020; Weemstra et al. 2017). Roots with a small diameter are, for instance, expected to scavenge for water resources in small water-filled soil pores; however, their development may be affected by an unfavorably dense soil structure that impedes the formation of this type of roots (Clark et al. 2003).

Environmental conditions that affect the distribution, concentration, and diffusivity of soil water also alter the metabolism of soil microbial communities (Brangarí et al. 2021; Clemmensen et al. 2006; Fuchslueger et al. 2014; Malik and Bouskill 2022; Schimel et al. 2007; Spohn and Chodak 2015; Tecon and Or 2017). In particular, the intensity of water stress can influence microbes’ ability to keep hydrated and utilize available C resources (Boot et al. 2013; Kakumanu et al. 2013; Schimel et al. 2007; Schimel 2018). In addition to direct physical effects, a lack of soil water has been observed to affect soil microbes through changes in substrate supply (Bardgett et al. 2008; Hartmann et al. 2017). Changes in plant C allocation belowground can, for instance, affect the quality and quantity of C available for soil microorganisms tightly connected to recently assimilated plant C, such as soil fungi and bacteria. Despite this improved understanding, the impact of progressing water loss on the transfer of assimilated C to root systems and associated soil microorganisms remains quantitatively unresolved (Joseph et al. 2020; McDowell et al. 2022; Prescott et al. 2020).

The C allocation within saplings is best assessed by the pulse labelling of their aboveground biomass with ^13^C enriched CO_2_ (^13^C-CO_2_) and by tracing of the newly assimilated ^13^C label in different aboveground and belowground compartments over repeated time points (Ruehr et al. 2009; Joseph et al. 2020). Compound-specific ^13^C isotope analysis of phospholipid fatty acid (PLFA) markers extracted from soils is an approach to determine the incorporation of the ^13^C label by specific soil microbial groups (Karlowsky et al. 2018; Kramer and Gleixner 2006). The ^13^C label released as CO_2_ from the respiration of living roots and soil organisms in the soil pore space can be measured in collected soil pore gas samples (Van de Broek et al. 2020).

The main aim of this study was to assess how increasing levels of water limitation affect tree growth and the allocation of newly assimilated C to roots and soil microorganisms. We investigated these processes in mesocosms featuring three-year-old Scots pine saplings and natural soil from a mature forest stand affected by reoccurring drought episodes. The mesocosms were exposed to different levels of water limitation. In late summer, at the end of the main growing season of the trees, we performed a ^13^C-CO_2_ pulse labelling and traced the pathway of assimilated C into tree needles, fine roots, soil pore CO_2,_ and phospholipid fatty acids of soil microbial groups. We hypothesized that increased water stress would reduce the magnitude of tree C uptake and the velocity at which newly assimilated C is transported belowground and further metabolized. Moreover, we expected that more severe levels of soil water deficit would lead to a build-up of newly assimilated C in fine roots.

## Material and methods

### Establishment of mesocosms

To study how different levels of water limitation affect the processes and interactions occurring at the interface between plants and soils, we established an experimental platform consisting of 18 Scots pine-soil systems at the greenhouse facility of the Research Station for Plant Sciences (ETH Zurich, Lindau, Switzerland), in September 2019. Each Scots pine-soil system (subsequently referred to as ‘mesocosm’) was set up by transplanting a three-year-old Scots pine sapling (*Pinus sylvestris* L, seed origin: Leuk, Switzerland, 980–1250 m a.s.l., with a mean height of 61 ± 1 cm and a mean stem diameter of 21 ± 1 mm) in a pot with the size of 32 cm height × 69 cm diameter (100 L volume). The pots were filled with a 2–3 cm bottom layer of stones (10–15 kg) to facilitate drainage of soil water and 20 cm of natural soil (100–110 kg). The soil (a Pararendzina developed from an alluvial fan and debris cone of the Ill river (Brunner et al. 2009; Guidi et al. 2022)) and the stones were collected at the margins of a xeric forest in the Rhone Valley, below the forest canopy (Pfywald, Canton Valais, Switzerland, 46°18′16.1″N, 7°36′44.8″E, 600 m a.s.l.). Details on the soil and trees used in the mesocosm experiment are provided in Methods S1.

### Irrigation treatments and temperature settings

**Fig. 1:**
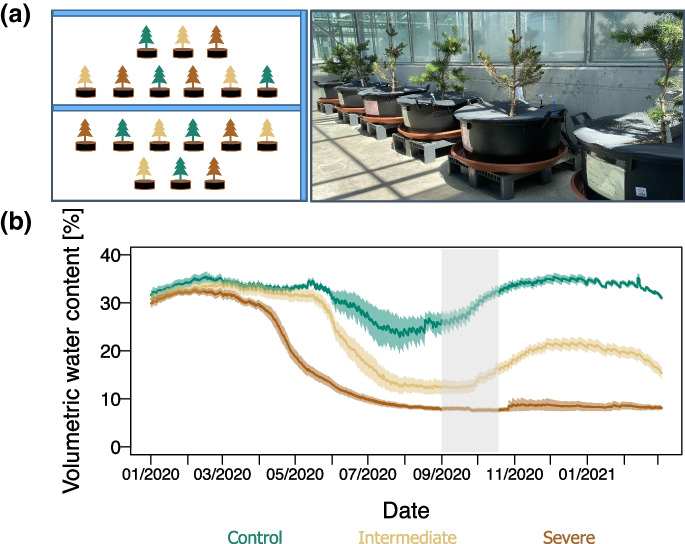
**a**) Experimental set-up of the Scots pine-soil mesocosms in the greenhouse (adapted from Jaeger et al. (2023)), **b**) Volumetric water content in % measured continuously in each of the 18 mesocosms. The lines represent the hourly volumetric water content data averaged across mesocosms and the shaded bands show the standard error (*n* = 6). Control (green), intermediate water limitation (dark yellow), severe water limitation (brown)

From September 2019 to January 2020, the mesocosms were watered with 2 L of local rainwater twice per week, reaching a volumetric water content (VWC) of approximately 30% (close to the field capacity of the soil: pF 1.8 ~ 35 VWC %). The water limitation experiment began in January 2020 by exposing the mesocosms to three different irrigation treatments (six mesocosms per treatment) in a randomized design to minimize spatial effects (i.e. variability in shading) (Fig. 1a). The amount of water to be supplied was controlled through automated soil moisture measurements (as detailed in Methods S2), according to the following treatments: Control, the mesocosms were supplied with sufficient soil water (close to field capacity, ca. 30% VWC; *n* = 6); Intermediate water deficit, the mesocosms were supplied with a moderately decreased amount of soil water (40% reduction in the amount of water supplied as compared to control; *n* = 6); Severe water stress, the mesocosms were supplied with a strongly decreased amount of soil water (75% reduction in the amount of water supplied as compared to control; *n* = 6). The intermediate water limitation treatment represents the maximum forecasted deviation of precipitation from the normal climate (1981 – 2010) for emission scenario RCP 8.5 in Southern Switzerland (NCCS 2018).

To ensure an exhaustive assessment of the soil moisture levels within the mesocosms, the gravimetric water content (GWC) of the soils was measured on a seasonal basis. For the latter, soil samples were collected using a stainless-steel auger with a 5.5 cm inner diameter down to 20 cm soil. These soil samples were additionally used for assessing concentrations of K_2_SO_4_ extractable organic C (EOC), as described in Methods S3.

The temperature conditions in the greenhouse were set to account for the seasonal changes in mean temperatures according to the climatological data measured at the meteorological station in Sion (Canton Valais, Switzerland) (MeteoSwiss, online dataset n.d.) (Table S1). The Sion meteorological station is located nearby the xeric forest dominated by Scot pine trees from which the soil for the mesocosms was collected.

### Aboveground tree growth and leaf gas exchange

Throughout the experiment, the height, leader shoot, and stem diameter of the Scots pine trees were monitored monthly (as detailed in Methods S4). On a seasonal basis, leaf gas exchange (light-saturated photosynthesis (A_net_) and stomatal conductance (g_s_)) were measured using a LiCor 6400 system (LI-COR Biosciences, Lincoln, NE, USA). Specifically, 25 south exposed needles were enclosed in the 2 × 3 cm chamber, and A_net_ and g_s_ were measured under 400 μmol mol^−1^ CO_2_, 1000 PAR, local humidity and temperature, and a stomatal ratio of 1. At the end of the first growing season of the trees in the mesocosms (on August 28^th^ 2020), predawn leaf water potential (Ψ) was measured on current-year twigs between 04:00 and 05:30 a.m., using a Scholander-type pressure chamber (PMS Instrument Company, Albany, NY, USA) in steps of 0.05 MPa. The leaf area of the needles was also determined at the end of the first growing season of the trees in the mesocosms by randomly collecting 30—40 needles throughout the whole crown of the trees and scanning them with a flatbed scanner (EPSON Expression 11000XL, EPSON, Suwa, Nagano, Japan). The scanned images were analyzed with the WinRHIZO program (version 2013, Regent Instruments Inc., Chemin Sainte-Foy, Quebec, Canada) to determine the leaf area of the needles (Albaugh et al. 2020), which was normalized per needle.

### ^13^C-CO_2_ pulse labelling

The allocation of photosynthetic assimilates to roots and soil microbes was followed by ^13^C-CO_2_ pulse labelling in nine randomly selected mesocosms (n = 3 per treatment, thereby with similar soil moisture conditions) at the end of the first main growing season of the trees (on September 2^nd^ 2020, Fig. S1). To avoid diffusion of ^13^C-CO_2_ in the soil matrix, the soil was covered with plastic foil before pulse labelling (Fig. S2). The plastic foil was sealed to the stem of the trees with plasticine. The aboveground portion of the Scots pine tree in each mesocosm was covered with a transparent plastic bag placed over a cylindrical chamber with a volume of 73 L (Fig. S2). The plastic bag was sealed with a cotton cord around the tree stem to ensure gas tightness. The ^13^C-CO_2_ labelling of each tree lasted 45 min and was done on the same day for all mesocosms between 08:30 and 12:30 am. A fan inside the chambers ensured air circulation. During the pulse labelling, we added 40 mL of ^13^C-CO_2_ (99.54 atom% ^13^C; Eurisotop, Saint-Aubin, France) to achieve CO_2_ with an isotopic composition of roughly 50 atom-% and a mixing ratio of about 1500 ppm. The latter was assessed during the ^13^C-CO_2_ labelling by collecting air samples from each chamber. The air samples were taken with a 60 ml syringe connected to an outlet port linked to a tubing inserted in the middle of the chamber. They were measured for CO_2_ concentration and C isotope composition as described in Methods S5. Each tree was illuminated with an additional halogen floodlight during the ^13^C-CO_2_ labelling to ensure high radiation and comparable light conditions.

### Sample collection following pulse labelling

Tree needles were collected one day before and 45 min, 1, 2, 3, 7, 14, and 47 days after pulse labelling. At each sampling time, 15 needles were collected randomly from the crown of the young Scot pine trees. The collected needles were immediately put in liquid nitrogen to interrupt any metabolic activity and, after transport to the laboratory, dried at 70 °C.

Soil samples were collected one day before and 1, 2, 3, 7, 14, and 47 days after pulse labelling. The soils were sampled around the stem of the trees at a 15 cm distance down to 20 cm soil depth using a stainless-steel auger with a 2 cm inner diameter. Soils were transported back to the laboratory on ice packs and immediately sieved through a 4 mm mesh. Fresh soil samples were used for GWC assessments, as described in Methods S3. Soil for phospholipid fatty acid (PLFA) analysis was frozen after sieving and stored at -20 °C until further preparation.

Roots were carefully picked out of the soil, and fine roots with a diameter < 2 mm were washed with Milli-Q water to remove any adhering soil particles. Dead roots were removed from the < 4 mm sieved soil samples based on qualitative visual characteristics such as colour and breakability (Solly et al. 2013). Living fine roots were dried at 70 °C and their dry weight was assessed.

Soil pore gas sampling was performed one day before the pulse labelling, and 1.5, 3 h, 1, 1.5, 2, 3, 5, 7, 14, 26, and 47 days after pulse labelling. A 60 ml syringe was used to collect soil pore gas samples from the outlet port described in Methods S2. For each soil pore gas sample, two 20 ml subsamples were transferred to pre-evacuated Labco exetainers (12 mL) for measurements of CO_2_ concentration and C isotopic composition.

### Carbon isotopic composition of needles, roots, and soil pore CO_2_

The dried needles and fine roots were weighed, milled, and placed into tin capsules to measure the C isotopic composition. *δ*^13^C values and total C concentrations of ground needle and fine root material were analysed at the Stable Isotope Facility of the University of California, Davis (UC Davis, CA, USA) using an Elementar Vario MICRO cube elemental analyzer (Elementar Analysensysteme GmbH, Langenselbold, Germany) interfaced to a Sercon Europa 20–20 isotope ratio mass spectrometer (Sercon Ltd., Cheshire, United Kingdom).

The collected soil pore gas samples were used to determine the concentration and the C isotopic composition of CO_2_ in the soil of the mesocosms using gas chromatography (456-GC, Bruker, Billerica, USA) and an isotope ratio mass spectrometer (Delta^plus^XP, Finnigan MAT, Bremen, Germany), respectively (as detailed in Methods S5).

### Extraction and measurement of phospholipid fatty acids

To determine the uptake of ^13^C label by different soil microbial groups, phospholipid fatty acid (PLFA) analysis was performed with a ﻿modified Bligh-Dyer method following Frostegård et al. (1991), based on protocols by Waldrop and Firestone (2006) and Zosso and Wiesenberg (2021), with some modifications as described in Methods S6. The PLFAs were separated, quantified, and identified on a Trace 1300 GC (Thermo Fisher Scientific, USA) equipped with a mass spectrometer, while the *δ*^13^C values of individual PLFA were determined using IRMS as detailed in Methods S7.

The fatty acids C14:0, C15:0, C16:0, C17:0, C18:0, and C20:0 were used as general bacterial biomarkers (Bligh and Dyer 1959). The biomarkers i15:0, a15:0, i16:0, i17:0, and a17:0 were used to identify gram-positive bacteria (Pennanen et al. 1999). The fatty acids 16:1ω7, 16:1ω5, cy17:0, 18:1ω7, and cy19:0 were used as biomarkers for gram-negative bacteria (Zogg et al. 1997). Gram-positive, gram-negative, and general bacterial markers were summed to total bacterial PLFA (Frostegård and Bååth 1996). To identify Actinobacteriota, the fatty acids 10Me16:0, 10Me17:0, and 10Me18:0 were used (Kroppenstedt 1985). 18:2ω6,9 was used as a marker of fungi (Federle et al. 1986). The fungal to -bacterial PLFA ratio was calculated by dividing the fungal biomarker by all the bacterial biomarkers. The ratio of gram-positive to gram-negative bacteria was calculated by dividing the sum of gram-positive bacteria by the sum of the gram-negative bacteria.

### Data analyses

The C isotopic composition is expressed in *δ* notation (‰) relative to the VPDB standard.

The ^13^C added by pulse labelling in the different tree and PLFA compartments (^*13*^*C excess allocated*, expressed as mg m^−2^) was computed as follows (Eq. 1):

1 $${}^{13}C\;excess\;allocated={}^{13}C\;enrichment\ast Cpool$$

where the ^13^C enrichment represents the relative abundance of ^13^C enrichment above the natural C isotope. To calculate the relative abundance of ^13^C enrichment above the natural C isotope, the *δ* values were converted to *atom%* with Eq. 2:

2 $$atom\%=\left(\frac{100}{\frac{1}{\left(\frac{\delta }{1000}+ 1\right)*0.0111802} +1}\right)$$

where 0.0111802 is the accepted C isotope ratio of VPDB.

The ^13^C enrichment was calculated with Eq. 3:

3 $${}^{13}C\;enrichment=\frac{atom\%l-atom\%n}{100}$$

where *atom% n* is the natural ^13^C/^12^C background (in atom %) of an aboveground or belowground compartment before pulse labelling, and *atom% l* describes the ^13^C/^12^C ratio of the same compartment at a given time point after the ^13^C-CO_2_ pulse labelling. The *C pool* of roots and needles represents the total weight of these tree compartments (as detailed in Methods S8), expressed as mg dry weight m^−2^ ground area of the mesocosms (each mesocosm had a ground area of 0.37 m^−2^) and multiplied by the percentage of C in the samples and divided by 100. The *C pool* of the individual PLFAs (expressed as mg PLFA in soil dry weight m^−2^ ground area) represents the PLFAs’ concentration per dry weight soil mass within the ground area (0–20 cm depth).

To calculate the ^13^C released as soil pore CO_2_ after pulse labelling (^*13*^*C excess released,* expressed as mg ^13^C m^−2^ h^−1^*),* we used the following equation (Eq. 4):

4 $${}^{13}C\;excess\;released={}^{13}C\;enrichment\ast{CO}_2F$$

where the *CO*_*2*_* F* represents the modelled CO_2_ efflux (expressed as mg C m^−2^ h^−1^). A description of how the CO_2_ efflux was modelled is provided in Methods S9.

The fraction of ^13^C label transferred to different belowground compartments was calculated as the ratio of the ^13^C excess in the compartment relative to the total ^13^C label assimilated by the trees. The latter was assessed for each mesocosm by measuring the C isotopic composition and calculating the ^13^C excess in the needles 45 min after the end of the pulse labelling.

We estimated the mean residence time of the ^13^C label in the Scots pine needles by using the following exponential decay function (Eq. 5) as in (Ruehr et al. 2009):

5 $$N\left(t\right)={N}_{o}{e}^{-\lambda t}$$

*N(t)* denotes the ^13^C excess at time *t*, N_o_ the ^13^C excess at the labelling peak, and λ is the decay constant. The mean residence time was then calculated as $\tau =1/\uplambda$. The time lag of the ^13^C signal appearing in soil pore CO_2_ related to the height of the trees was calculated to provide an estimate of the velocity of the stem transport of newly fixed assimilates to the rhizosphere (including roots and soil microorganisms) (Gao et al. 2021).

### Statistics

We conducted statistical analyses with R, Version 4.2.1 (R Core Team 2022). Throughout the manuscript, we present data as mean ± standard error. For all statistical tests, we used a significance level of 0.05. We transformed the continuous variables to normal distributions when required to meet the normality assumptions of the applied statistical tests. To test the effect of the irrigation treatments and sampling times on the measured tree and soil parameters, we used the linear mixed effect function of the package *nlme* v. 3.1–1588 (Pinheiro et al. 2022) with the restricted maximum likelihood method *‘REML’* (Meyer 1989). Treatment and sampling time (season) were considered fixed effects, and pot and greenhouse were random effects for GWC, soil temperature, A_leaf_, g_s_, tree height increment, stem diameter increment, and EOC. Treatment was considered a fixed effect, and pot and greenhouse were random effects for parameters measured at the time of ^13^C-CO_2_ pulse labelling, i.e., Ψ_,_ leader elongation, needle area, needle biomass, fine root length and diameter, fine root biomass and fraction of root biomass (root biomass / total tree biomass), soil pore CO_2_ concentrations and modelled CO_2_ effluxes, as well as concentrations of individual PLFAs. We checked the model assumptions using the diagnostic plot functions (Crawley 2012), and the normality of the residuals was tested with histograms. To test the effect of treatment and sampling time (season) on soil GWC pairwise comparisons were estimated using marginal means adjusted with the Tukey method, with the package *emmeans* v. 1.8.1–1 (Lenth et al. 2022). We used linear least-squares to compare correlations between the GWC and VWC of the soil as well as the soils’ GWC and EOC. Regression analyses were used to assess the relationships between the parameters illustrated in Fig. 7. Correspondence analysis was employed to obtain graphical representations of the variability in the mean relative abundance of individual PLFA markers in relation to water limitation across all sampling times, using the package *ca* v. 0.71.1 (Greenacre et al. 2018).

## Results

### Experimental soil water

The VWC decreased steeply after the start of the irrigation treatments in January 2020 (control, intermediate water limitation, and severe water limitation) (Fig. 1b). The three different water regimes reached the anticipated level in spring 2020. Throughout the experiment, the VWC was well correlated to the GWC of the soils (R^2^ = 0.96, P < 0.001, Fig. S3a). The GWC of the soil differed significantly among the three treatments, with the lowest values in summer and autumn 2020 (Tables 1 and 2). At the end of summer 2020, when we traced the fate of the ^13^C-CO_2_ pulse label through the plant-soil systems, the GWC of the control treatment was larger and had a greater variability (ranging between 32.7 – 14.3%) as compared to that of the intermediate (12.2 – 4.7%) and the severe (5.5 – 1.9%) water-limiting treatments (Dataset S1).

**Table 1:** Seasonal gravimetric water content in the soils of the mesocosms during the experiment. Means ± standard errors (n = 6) are presented for each irrigation treatment. Small letters indicate significant differences between groups based on estimated marginal means and adjusted with the Tukey method

Treatment	Winter 20	Spring 20	Summer 20	Autumn 20	Winter 21
Control	33.5 ± 0.5^a^	36.2 ± 3.0^a^	18.0 ± 3.1^bc^	27.5 ± 1.2^a^	33.9 ± 0.8^a^
Intermediate	32.8 ± 0.7^a^	25.4 ± 1.6^ab^	12.3 ± 3.9^c^	10.2 ± 0.7^c^	13.8 ± 0.7^c^
Severe	31.6 ± 0.6^a^	9.9 ± 0.9^c^	4.7 ± 0.2^d^	3.8 ± 0.1^d^	4.7 ± 0.4^d^

**Table 2:** Outputs of linear mixed-effects models testing the effect of experimental treatments and seasonal sampling times on gravimetric water content (GWC), soil temperature, light-saturated photosynthesis (A_net_), stomatal conductance (g_s_), tree height increment, stem diameter increment and soil-extractable organic carbon (EOC). Numbers in bold represent significant effects (P < 0.05)

Parameter	Unit	Treatment		Sampling time (season)		T x S	
		F	*P*	F	*P*	F	*P*
GWC	[%]	**141.01**	** < 0.001**	**53.69**	** < 0.001**	**11.73**	** < 0.001**
Soil temperature	[°C]	2.524	ns	**2811.63**	** < 0.001**	0.796	ns
A_net_	[µmol m^−2^ s^−1^]	**33.85**	** < 0.001**	**297.29**	** < 0.001**	**7.87**	** < 0.001**
g_s_	[ mol m^−2^ s^−1^]	**71.35**	** < 0.001**	**5.944**	** < 0.001**	**5.797**	** < 0.001**
Tree height	[cm]	0.690	ns	**42.390**	** < 0.001**	0.593	ns
Stem diameter	[mm]	0.08	ns	**25.15**	** < 0.001**	0.05	ns
EOC	[ug C g^−1^ soil]	2.479	ns	**28.063**	** < 0.001**	**3.411**	** < 0.01**

### Tree gas exchange

**Fig. 2:**
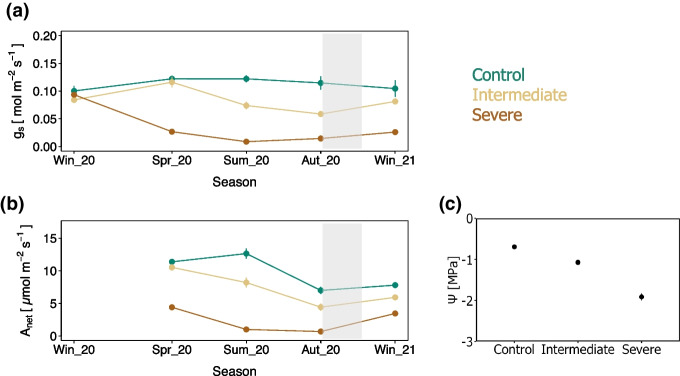
Seasonal changes in **a**) stomatal conductance (g_s_), **b**) light-saturated photosynthesis (A_net_), and **c**) visualization of the difference in predawn leaf water potential (Ψ) among treatments at the time of ^13^C-CO_2_ pulse labelling. In panels a and b, the shaded background represents the period during which the ^13^C pulse was traced in the mesocosms. The means ± standard errors (*n* = 6) are presented. Control (green), intermediate water limitation (dark yellow), severe water limitation (brown)

In response to water limitation, A_net_ and g_s_ decreased significantly for trees growing under water stress compared to control trees (Table 2, Fig. 2). The response to water limitation was faster for trees growing under severe water stress than those growing under intermediate water limitation. For instance, only the trees growing under severe water deficit presented a steep decline in A_net_ and g_s_ in spring 2020, while the gas exchange parameters of trees growing under intermediate water deficit were not significantly reduced until summer 2020 (Fig. 2a, b). In summer 2020, just before the ^13^C-CO_2_ pulse labelling took place, the predawn leaf water potential Ψ was significantly larger for the control trees, followed by that of the trees growing under intermediate water limitation and severe water limitation (Fig. 2c, Table. 3).

**Table 3:** Outputs of linear mixed-effects models testing the effect of experimental treatments, at the time of ^13^C-CO_2_ pulse labelling, on predawn leaf water potential (Ψ), leader elongation, needle area (normalized per needle), needle and fine root biomass per ground area, fine root length per soil core volume, mean fine root diameter, and the fraction of root biomass (root biomass / total tree biomass). Numbers in bold represent significant effects (*P* < 0.05)

	Unit	Treatment	
Parameter		F	P
Ψ	[MPa]	**42.41**	** < 0.001**
Leader elongation	[cm]	0.32	ns
Needle area	[cm^2^]	**26.33**	** < 0.001**
Needle biomass	[g m^−2^]	**26.59**	** < 0.001**
Fine root biomass	[g m^−2^]	0.725	ns
Fine root length	[cm cm^−3^]	**10.53**	** < 0.01**
Fine root diameter	[mm]	**4.583**	** < 0.05**
Fraction of root biomass	[g g^−1^]	**6.517**	** < 0.01**

### Tree aboveground and belowground growth

**Fig. 3:**
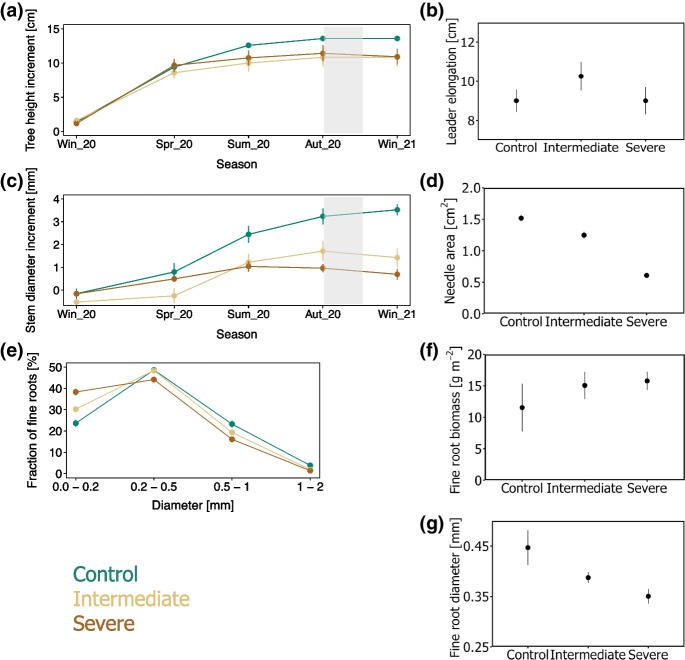
Seasonal changes in **a**) tree height increment, **b**) visualization of the difference in leader elongation among treatments at the time of ^13^C-CO_2_ pulse labelling, **c**) seasonal changes in tree diameter increment, **d**) visualization of the difference in needle area (normalized per needle) at the time of ^13^C-CO_2_ pulse labelling, **e**) fraction of fine root length in different root diameter sizes at the time of ^13^C-CO_2_ pulse labelling, **f**) visualization of the difference in fine root biomass (expressed in g m^−2^ per ground area) at the time of ^13^C-CO_2_ pulse labelling, **g**) visualization of the difference in mean diameter of fine roots at the time of ^13^C-CO_2_ pulse labelling. In panels a and c, the shaded background represents the period during which the ^13^C pulse was traced in the mesocosms. In all panels the means ± standard errors (*n* = 6) are presented. Control (green), intermediate water limitation (dark yellow), severe water limitation (brown)

The main increase in tree height occurred during the spring when the trees grew on average 10 ± 1 cm between March and May 2020 (Fig. 3a, Table 2), with no significant difference in leader elongation among the three irrigation treatments (Fig. 3b, Table 3). The stem diameter of the trees mainly increased between spring 2020 and autumn 2020, with a larger stem diameter increment observed for the control trees as compared to the trees under water deficit (Fig. 3c, Table 2). At the end of the main growing season of the trees, when the ^13^C-CO_2_ pulse labelling took place, the needle area of the control trees and trees growing under intermediate water limitation was significantly larger than that of severely water-limited trees (Fig. 3d, Table 3).

The production of fine root biomass did not significantly differ among the three levels of irrigation (Fig. 3f). Nevertheless, the morphological analysis of fine root samples at the end of the main growing season indicated significant changes in fine root traits (Table 3). We observed that the overall fine root length per soil volume was significantly greater for the trees growing under severe water deficit (1.79 ± 0.31 cm cm^−3^) as compared to the fine roots of trees growing under intermediate water deficit (0.99 ± 0.29 cm cm^−3^) and control conditions (0.50 ± 0.25 cm cm^−3^) (Dataset S2). The average diameter of the fine roots was significantly smaller for the severely water-limited trees as compared to the fine roots of trees growing under intermediate water deficit and control conditions (Fig. 3g). The proportion of fine roots recovered in different diameter sizes varied among the three treatments (Fig. 3e).

Moreover, Table 4 shows that the total tree biomass and needle biomass at the time of pulse labelling were lower for the severe water limitation treatment in comparison to the other irrigation treatments. The root biomass fraction (root biomass / total tree biomass) was instead largest under severe levels of water deficit.

**Table 4:** Differences in total tree biomass, needle biomass, root biomass fraction (root biomass / total tree biomass), tree height, and tree diameter among the three irrigation treatments at the time of ^13^C-CO_2_ pulse labelling. Means ± standard errors are presented (*n* = 6) for each irrigation treatment

	Unit	Control	Intermediate	Severe
Total tree biomass	[g dry weight]	193 ± 271	170 ± 9	168 ± 7
Needle biomass	[g dry weight]	35 ± 1	34 ± 1	25 ± 1
Root biomass fraction	[g g^−1^]	0.15 ± 0.02	0.26 ± 0.03	0.30 ± 0.02
Tree height	[cm]	72 ± 3	72 ± 3	72 ± 3
Tree diameter	[mm]	25 ± 2	22 ± 1	24 ± 1

### Extractable soil organic carbon

Although the amount of soil-extractable organic C (EOC) did not significantly differ among the three treatments (Table 2), it significantly varied among sampling times, with the greatest values observed during winter and spring (Fig. S3b).

### Dynamics of ^13^C allocation

#### Needles

**Fig. 4:**
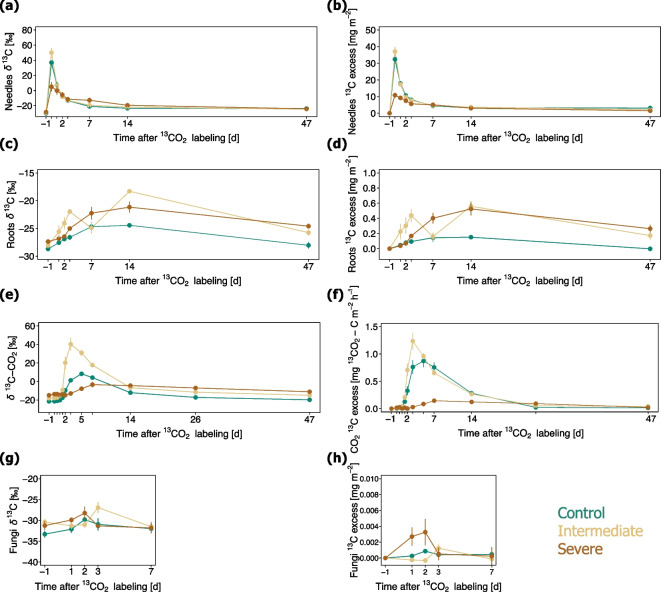
Dynamics of *δ*^13^C and ^13^C excess in Scots pine needles (**a**, **b**), fine roots (**c**, **d**), soil pore CO_2_ (**e**), modelled soil CO_2_ efflux (**f**), and fungal PLFA (**g**, **h**). The means ± standards error (*n* = 3) are presented. Control (green), intermediate water limitation (dark yellow), severe water limitation (brown)

 At the first sampling point of the Scots pine needles, 45 min after the end of the ^13^C-CO_2_ pulse labelling, the ^13^C excess in the needles ranged between 7.5 and 45.8 mg^13^C m^−2^, demonstrating that all trees assimilated a significant portion of ^13^C during the pulse labelling (Fig. 4a, b). The amount of ^13^C excess detected in the needles of trees exposed to intermediate water deficit (37.1 ± 5.2 mg^13^C m^−2^) was in a similar range as that of the control trees (32.4 ± 2.4 mg^13^C m^−2^), while the trees exposed to severe water deficit carried a markedly lower ^13^C excess (10.8 ± 2.5 mg^13^C m^−2^). The mean residence time of the ^13^C label in the needles was calculated to be longer for trees growing under severe water deficit (9.3 ± 0.7 days) as compared to trees growing under intermediate water limiting conditions (3.6 ± 0.4 days) and control trees (3.4 ± 0.1 days).

#### Fine roots

The ^13^C label measured in fine roots appeared one day after pulse labelling (Fig. 4c, d). The label was still detectable in the fine roots of the trees 47 days after pulse labelling, shifting the δ^13^C values by 2 ± 0.6‰ from the ambient levels measured one day before ^13^C-CO_2_ pulse labelling. Throughout all sampling times, the ^13^C excess measured in the fine roots of the Scots pine trees remained greater for the trees growing under intermediate and severe water limitation, as compared to the control trees (Fig. 4d).

#### PLFAs

An increased allocation of ^13^C label to fungal PLFA was observed under conditions of severe water scarcity (Fig. 4g). The ^13^C excess in the fungal lipid marker was observed to reach a maximum value two days after pulse labelling (Fig. 4h). The incorporation of ^13^C label by the other individual lipid markers was very low under control and water-limiting treatments (Dataset S3).

**Fig. 5:**
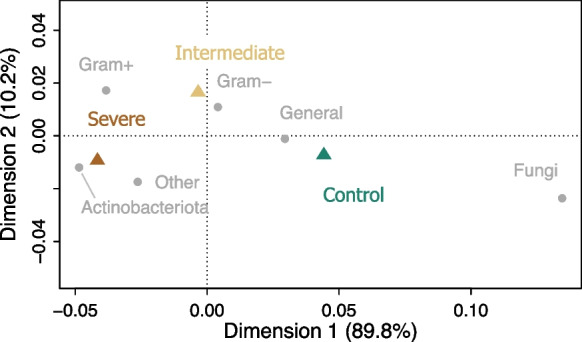
Correspondence analysis visualizing the variability in the mean relative abundance of individual PLFA markers representing different groups of soil microbes in relation to the three irrigation treatments (control, intermediate water limitation, severe water limitation). The mean relative abundance of the individual PLFA markers was calculated across all sampling times (*n* = 15)

Regarding biomass, none of the microbial PLFAs were significantly influenced by water limitation (Table 5). Nevertheless, throughout all sampling dates the Actinobacteriota presented a marginal increase in abundance under intermediate (0.63 ± 0.04) and severe (0.76 ± 0.07) water limitation as compared to the control (0.57 ± 0.04) (Table 5). Moreover, the fungal to bacterial PLFA ratio was observed to be significantly lower under the intermediate (0.030 ± 0.003) and severe (0.028 ± 0.001) water-limiting treatments as compared to the control (0.038 ± 0.003) when averaged across all sampling times during the pulse labelling experiment (Table 5). As displayed by the correspondence analysis, soil microbial groups responded with contrasting sensitivity to reduced levels of soil moisture (Fig. 5).

**Table 5:** Outputs of linear mixed-effects models testing the effect of the three irrigation treatments on the phospholipid fatty acids (PLFAs), which were used as biomarkers for different microbial groups, and of the soil pore CO_2_ concentrations and across the course of the ^13^C-CO_2_ pulse labelling. Numbers in bold represent significant effects (P < 0.05)

	Treatment	
PLFA [ug g^−1^dw soil]	F	*P*
Total	1.337	ns
General bacteria	0.544	ns
Gram-positive	1.681	ns
Gram-negative	0.948	ns
Actinobacteriota	2.871	ns* (0.068)
Fungi	1.446	ns
Fungi: bacteria	**4.15**	**0.0227**
Gram-positive: Gram-negative	1.864	ns
Soil pore CO_2_ [ppm]	**131.3**	** < 0.001**
CO_2_ efflux [gC m^−2^ h^−1^]	**5.04**	** < 0.01**

#### Soil pore CO_2_

While tracing the ^13^C-CO_2_ pulse label, the CO_2_ concentrations measured in soil pores were significantly higher for soils kept under control conditions (4079 ± 274 ppm), as compared to soils affected by intermediate (1122 ± 32 ppm) and severe water limitation (737 ± 17 ppm) (Table 5). The modelled CO_2_ effluxes based on temperature and moisture dependencies applied to continuously monitored soil temperatures and VWC also significantly differed among irrigation treatments during the ^13^C-CO_2_ labelling experiment (Table 5). The ^13^C pulse added to the crowns of the trees started to appear in the soil pore CO_2_ after 1.5 days for the control and intermediate water limitation treatment, and after 3 days for the severe water limitation treatment (Fig. 4e). The level of water limitation influenced the temporal dynamics of the modelled ^13^C excess in the soil CO_2_ effluxes (Fig. 4f). The ^13^C excess of the control treatment reached a maximum five days following pulse labelling (Fig. 4f), that is 2 days after the peak of the intermediate water-limiting treatment and 2 days before the peak of the severe water-limiting treatment. The average transport velocity of newly assimilated ^13^C label from Scot pine needles to the soil-respired CO_2_ through the stem of the trees was comparatively faster under control (46 ± 2 cm day^−1^) and intermediate conditions of water limitation (44 ± 2 cm day^−1^) than under conditions of severe water deficit (25 ± 2 cm day^−1^).

### Fraction of ^13^C label allocated to different aboveground and belowground compartments

**Fig. 6:**
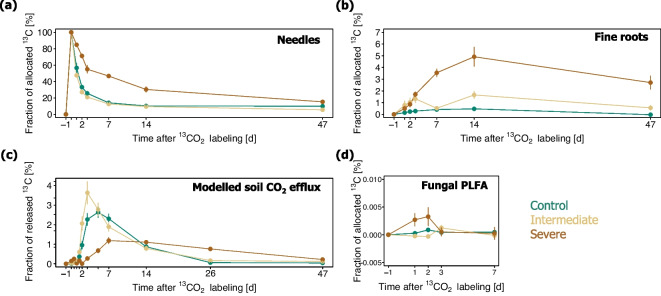
Fraction of ^13^C label allocated to **a**) needles, **b**) fine roots, **c**) released through the modelled soil CO_2_ efflux, and d) fungal PLFA. The fractions were calculated by normalizing the ^13^C excess of the various C pools by the total amount of ^13^C label assimilated by the needles of the Scots pines during ^13^C-CO_2_ pulse labelling. The means ± standard errors (*n* = 3) are presented. Control (green), intermediate water limitation (dark yellow), severe water limitation (brown)

**Fig. 7:**
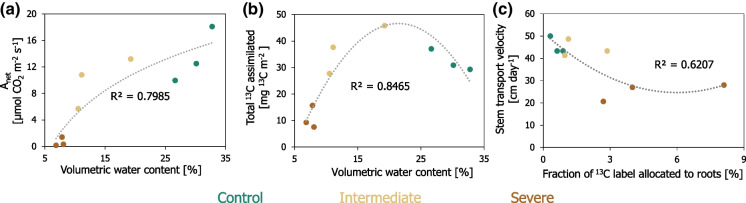
Relationship between **a**) volumetric water content and light-saturated photosynthesis (A_net_) at the time of ^13^C-CO_2_ pulse labelling, **b**) volumetric water content and total assimilation of ^13^C label in Scots pine needles during ^13^C-CO_2_ pulse labelling, and **c**) the maximal fraction of ^13^C label allocated to fine roots after ^13^C-CO_2_ pulse labelling and the average transport velocity of the ^13^C label from Scot pine needles to the soil-respired CO_2_ along the stem of the trees. Lines represent best fits to logarithmic (panel a) and polynomial (panels b and c) functions. Control (green), intermediate water limitation (dark yellow), severe water limitation (brown)

To assess how the different levels of water limitation affected the fraction of newly assimilated ^13^C label allocated to belowground C compartments, we divided the ^13^C excess of the different belowground C compartments by the maximum ^13^C excess measured in the needles of the trees (i.e., the ^13^C excess assessed in the needles 45 min after the end of the pulse labelling, the highest values in Fig. 4b). It emerged that when compared to control conditions, the fraction of ^13^C label allocated to fine roots and soil fungi increased with more severe conditions of water limitation (Fig. 6b, d). Moreover, the fraction of ^13^C label allocated to fine roots showed a negative relationship with the transport velocity of the ^13^C label from Scot pine needles to the soil-respired CO_2_ along the stem of the trees (Fig. 7c).

## Discussion

**Fig. 8:**
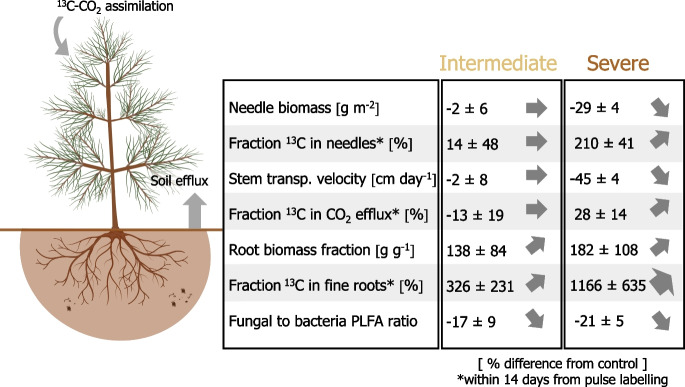
Summarizing scheme of the main results of this study. The fraction of ^13^C label allocated to needles, CO_2_ efflux, and fine roots within 14 days from ^13^C-CO_2_ pulse labelling was calculated by normalizing the ^13^C excess of the various C pools by the total amount of ^13^C label assimilated by the needles of the Scots pines during the labelling. Values are shown as [(water limitation treatment–control)/control*100]. The means ± standard errors (*n* = 3, except *n* = 6 for the needle biomass and the root biomass fraction) are presented. The variation was derived by calculating the percent difference between a water-limited sapling and the closest control sapling in the greenhouse. The arrows represent the direction of change. The image of the tree was created with BioRender.com

Our study showed that the allocation of newly assimilated C within Scots pine-forest soil systems depends on the levels of soil moisture (as summarized in Fig. 8). By assessing the growth of Scots pine saplings and tracing the pathway of newly assimilated ^13^C label into different aboveground and belowground compartments at the end of the growing season, we found that severe levels of water deficit strongly reduced the aboveground growth of the saplings and the magnitude of tree C uptake and the velocity at which newly assimilated C is transported belowground and further metabolized. In comparison, moderate levels of soil water limitation barely affected the cycling of C at the tree-soil interface. These findings are novel as the ecological implications underlying plant responses to drought still remain highly uncertain due to a lack of understanding of how individual tree species respond with abrupt changes in their physiology to different levels of water limitation (Walthert et al. 2021).

### Influence of water limitation intensity on the development of Scots pine saplings

The level of soil moisture strongly influenced the development of the Scots pine trees in the mesocosms. We observed that while the needle area and growth of the stem diameter decreased, the root biomass fraction (root biomass / total tree biomass) increased with more intense levels of water limitation (Figs. 3a, d and 8, Table 4). These changes in plant biomass partitioning support findings from previous studies showing that under low levels of soil moisture plants can maintain the proportion of roots to sustain water uptake and photosynthetic C assimilation at the expense of aboveground growth (Klein et al. 2011; McDowell et al. 2008; Oberhuber et al. 2011; Poorter et al. 2012). In agreement, we further observed significant alterations in fine root traits associated with resource scavenging, such as smaller fine root diameters and longer root lengths per soil volume (Fig. 3e, g, Table 3), maintaining tree vitality under reduced soil water levels (Comas et al. 2012). Longer term studies would help to understand whether trees subjected to prolonged or recurrent episodes of soil water limitation maintain an increased root biomass fraction over time or rather adjust to a lower water demand (related to a reduced aboveground biomass) by allocating less C to root growth (Bose et al. 2022).

### Carbon assimilation and transit time responses to water limitation intensity

In our study, severe levels of water deficit reduced the amount of C assimilated by the needles of the trees at the time of ^13^C-CO_2_ pulse labelling by approximately three times when compared to control trees (Fig. 7b). A lower assimilation of C is consistent with the low photosynthetic rate that we observed for trees affected by severe water limitation (Figs. 2b and 7a). Intermediate levels of water stress did not affect the uptake of C in comparison to control trees (Fig. 7b), despite a non-significant reduction in photosynthesis under intermediate water stress (Fig. 7a, P > 0.05). The higher CO_2_ concentrations during the pulse labelling might have compensated for stomatal closure induced by limited soil water (Morison 1985). However, in our study, the CO_2_ concentrations measured in the chambers during pulse labelling were similar for the three irrigation treatments, indicating that the influence of the higher CO_2_ concentrations during the pulse labelling on the amount of C assimilated by the trees was negligible.

When compared to the control, only severe water deficit prolonged the mean residence time of the ^13^C label in Scots pine needles and slowed down the transport velocity of newly assimilated C from needles to soil-respired CO_2_ (Fig. 8). The stem transport velocity of 25 cm day^−1^ (0.01 m h^−1^) in the Scots pine saplings growing under severe water stress is comparable to the published transport velocity of 0.01 m h^−1^ observed in beech saplings affected by drought (Ruehr et al. 2009), and slower as compared to mature trees (Dannoura et al. 2011). We attribute the longer transit time of C within trees affected by severe water limitation to a threshold of soil moisture at which the C metabolism of the trees was slowed down as well as to the markedly reduced photosynthetic rates (which likely prolonged the time needed to assimilate new C to dilute the ^13^C label assimilated in the needles). A few ^13^C tracer studies have already indicated that intense episodes of drought slowed down the mean transit time of newly assimilated C in plants (Barthel et al. 2011; Hasibeder et al. 2015; Joseph et al. 2020). Here, the Scots pines substantially slowed down their C transit time at soil moisture levels below 10% VWC (which for our soil are close to wilting levels).

### Belowground carbon allocation responses to water limitation intensity

Both intermediate and severe levels of experimental water limitation increased the magnitude of ^13^C excess in fine root systems (Figs. 4d and 8). A higher C allocation to roots under water stress is known as the ‘optimal partitioning theory’ after Bloom et al. (1985), which states that plants allocate more nutrients and C to belowground tissues when they are limited by water or nutrient shortage. In our study, the fraction of recently assimilated ^13^C label transported to the fine roots of control trees and trees growing under intermediate water deficit appeared to be rapidly metabolized and released through the modelled soil CO_2_ efflux (Fig. 6b, c). The fraction of ^13^C label, which was allocated to fine roots, was instead larger than the fraction of ^13^C released through the modelled soil CO_2_ efflux in the mesocosms treated with severe soil moisture stress (Fig. 6b, c). The comparatively larger fraction of assimilated ^13^C label detected in fine roots of severely stressed trees may be implied by an accumulation of C in the fine root system or a slower metabolization of C belowground. For instance, an increase in non-structural carbohydrates such as starch and sucrose and/or osmotic adjustments to water deficit have been observed in previous studies (Hasibeder et al. 2015; Prescott et al. 2020; Tang et al. 2022). The modelled soil CO_2_ efflux data from our study further indicated that, as compared to the control, the fraction of newly assimilated ^13^C label being released back to the atmosphere via soil respiration was marginally higher under intermediate water limitation but considerably lower under conditions of severe water deficit (Fig. 6c). This pattern is a further indication that the ^13^C label allocated belowground continued to be readily used for root (autotrophic) and microbial (heterotrophic) respiratory C metabolism in soils kept under moderate levels of soil water stress, while it was barely used for metabolic processes in soils affected by severe water limitation. A lower metabolic activity and potential accumulation of assimilates as non-structural carbohydrates in fine roots under severe water stress would support the concept that a reduced sink activity belowground controls the C balance of trees (Hagedorn et al. 2016; Joseph et al. 2020). Nevertheless, it should be considered that the allocation of C to root systems is also tightly related to the age, growth, and physiology of individual tree species (Gessler and Grossiord 2019; Ledo et al. 2018; Rog et al. 2021), as well as on the time in the year. For instance, at the end of the growing season, when we performed the ^13^C-CO_2_ pulse labelling experiment, more carbon may have been accumulated in fine roots and transferred to the soil and associated soil microorganisms rather than used for the formation of new root systems. In any case, starting from the 14^th^ day after pulse labelling onwards, a greater proportion of the assimilated ^13^C label was detected in the CO_2_ efflux of severely water-limited soils (Figs. 6c and 8). Although disentangling the respective respiratory activity of the autotrophic and heterotrophic sources was beyond the scope of our study, our results suggest that a severe lack of water resources may not only reduce the photosynthetic assimilation and transport of C belowground but also prolong the supply of newly assimilated C to the soil CO_2_ efflux.

While tracking the fate of the ^13^C-CO_2_ label in our mesocosms, we used PLFA biomarkers to assess the different uptake of ^13^C tracer among soil microbial groups. Although, on average, most individual markers did not incorporate significant amounts of ^13^C label for any of the treatments, possibly due to the low fine root biomass diluting the ^13^C signal, we observed that in comparison to the control, an increased ^13^C excess and uptake of ^13^C label by soil fungi occurred under water scarcity (Fig. 4g, h). This finding, and our observation that the soil EOC did not significantly differ among the three irrigation treatments, suggest that the amount of plant-derived organic C released belowground and taken up by soil fungi remains sustained. In a previous study, Fuchslueger et al. (2014) pointed to a continued transfer of C from plants to fungi under experimental drought. Our results also align with the hypothesis by Prescott et al. (2020) that the flux of photosynthates to roots and associated microbial organisms is sustained when aboveground growth is constrained. Nevertheless, the effects of water limitation on microbial metabolism are context-dependent (e.g., soil physicochemical properties, local temperatures, etc.) and are likely influenced by the strength and duration of the occurring episodes of stress (Karlowsky et al. 2018). Moreover, a different physiology and phenological stage of plants can lead to a diverse microbial use and availabilities of organic matter resources in soils (Pugnaire et al. 2019). It should be further specified that in our study the level of soil moisture in the mesocosms under the severe water limitation treatment was kept at a level at which the saplings received a minimum of water to remain vital. It is likely that a more acute soil water limitation would have led to a depletion of C pools (as recently reviewed by McDowell et al. (2022)), a phloem transport failure, and an impeded transport of C belowground.

### Effect of water limitation intensity on soil microbes

Water limitation did not alter the total PLFA microbial biomass, but reduced the ratio of fungal to bacterial PLFAs (Fig. 8, Table 5). As visualized in the correspondence analysis (Fig. 5), this reduction was likely related to a contrasting sensitivity of soil microbial groups to reduced levels of soil moisture. Since the biomarker 18:2ω6,9 in the soil was found to be highly correlated to ectomycorrhizal root colonization (Kaiser et al. 2010), the observed decrease in the ratio between fungi and bacteria with water limitation may point to a change in the abundance of ectomycorrhizal fungi against the background of bacterial groups. A potential reduction of symbiotic microorganisms of trees is in line with DNA-based assessments of the soil microbiome in the same mesocosm platform (Jaeger et al. 2023). However, Jaeger et al. (2023) also reported that other fungal groups were more resistant to changes in soil water contents. In any case, the ability of soil fungi to create large hyphal networks to scavenge for water and nutrients (Allen 2007; Hendrix et al. 1986) likely consented the sustained uptake of tree-derived ^13^C label in the mesocosms treated with soil water limitation. Our data further showed a marginal increase of Actinobacteriota in response to reduced soil water contents (Table 5). This suggests that Actinobacteriota might have accumulated in the soils affected by water deficit in our experiment (Jaeger et al. 2023), and is further evidence that this microbial group is stress-tolerant and may proliferate at low osmotic potential (Bouskill et al. 2013), and in water-limited Scots pine forest soils (Hartmann et al. 2017). Overall, our findings highlight that altered soil moisture conditions can shift the composition of microbial communities (Manzoni et al. 2012; Schimel et al. 2007; Strickland and Rousk 2010), despite not necessarily altering the total soil microbial biomass (Hartmann et al. 2017).

## Conclusions

Our experiment indicates that moderate levels of water deficit do not profoundly affect photosynthetic C assimilation and the transit time of C from needles to the rhizosphere. In contrast, more severe water limitation alters these dynamics. As soil water becomes less available, Scot pine saplings start reducing their aboveground growth and increase the fraction of newly assimilated C allocated belowground. Under moderate levels of water deficit, most of the C allocated belowground is readily metabolized. However, when soil water levels become very low, part of the C allocated belowground may accumulate in root tissues. Nevertheless, the flux of C from plants to fungi seems not to be interrupted, probably until trees suffer from permanent damage and phloem transport failure. Overall, our results suggest that long-lasting episodes of water deficit strongly slow down the cycling of C within trees. However, effects related to tree age should be considered, as in mature trees C allocation dynamics may differ from those of saplings due to larger C pools. Considering how different levels of soil water limitation shift C allocation dynamics within trees may help forecast tree functioning and the fate of assimilated C during episodes of water stress.

### Supplementary Information

Below is the link to the electronic supplementary material.

Supplementary file1 (DOCX 1160 KB)

Supplementary file2 Soil gravimetric water content (GWC), volumetric water content (VWC), and K_2_SO_4_ extractable organic carbon concentrations (EOC) at the time of seasonal soil sampling; and GWC, VWC, and mean daily temperature of soils during the ^13^C-CO_2_ pulse labelling experiment. (XLSX 19 KB)

Supplementary file3 Seasonal measurements of tree height and stem diameter increment, stomatal conductance (g_s_) and light-saturated photosynthesis (A_net_), predawn leaf water potential, (Ψ) needle area, fine root biomass and morphology at the time of ^13^C-CO_2_ pulse labelling. (XLSX 18 KB)

Supplementary file4 Carbon isotopic composition of tree needles, fine roots, soil pore CO_2_, and phospholipid fatty acids (PLFA); total C concentration in needles and fine roots; soil pore CO_2_ concentrations and data used for PLFA quantification during the ^13^C-CO_2_ pulse labelling experiment. (XLSX 63 KB)

## Data Availability

The data that support the findings of this study are provided in the Supplementary Information files.
