# Supplementary material for: Water limitation intensity shifts carbon allocation dynamics in Scots pine mesocosms
Source: Plant Soil. 2023 Jun 17;490(1-2):499–519. doi: 10.1007/s11104-023-06093-5 (PMC10533586; doi:10.1007/s11104-023-06093-5)
Supplement: Supplementary file 1 — Supplementary file1 (DOCX 1160 KB) [file 11104_2023_6093_MOESM1_ESM.docx]

**Supporting information**

Article title: Water limitation intensity shifts carbon allocation dynamics in Scots pine mesocosms

Authors: Emily F. Solly, Astrid C. H. Jaeger, Matti Barthel, Roland A. Werner, Alois Zürcher, Frank Hagedorn, Johan Six, Martin Hartmann

Author for correspondence:

*Emily F. Solly*

*Email:emily.solly@usys.ethz.ch*

The following Supporting Information is available for this manuscript:

**Fig. S1:** Pictures of the mesocosms taken at the end of the first growing season of the trees.

**Fig. S2:** Picture of Scots pine-forest soil mesocosms during the ^13^C-CO_2_ pulse labelling.

**Fig. S3:** Visualization of the relationship between VWC and GWC, and between EOC and GWC.

**Table S1:** Seasonal greenhouse air temperature and humidity during the course of the mesocosm experiment.

**Methods S1:** Details on the soil and trees used in the mesocosm experiment (continuation of method description)

**Methods S2:** Automated soil moisture and temperature sensors and soil pore gas sampling system (continuation of method description)

**Methods S3:** Gravimetric water content and concentrations of K_2_SO_4_ extractable soil organic carbon (continuation of method description)

**Methods S4:** Aboveground tree growth parameters (continuation of method description)

**Methods S5:** ^13^C isotopic composition of CO_2_ in gaseous samples (continuation of method description)

**Methods S6:** Extraction of Phospholipid Fatty Acids (continuation of methods description)

**Methods S7:** ^13^C measurements of Phospholipid Fatty Acids (continuation of methods description)

**Methods S8:** Total weight of tree biomass compartments (continuation of methods description)

**Methods S9:** Soil CO_2_ efflux (continuation of methods description)

**Dataset S1:** Soil gravimetric water content (GWC), volumetric water content (VWC), and K_2_SO_4_ extractable organic carbon concentrations (EOC) at the time of seasonal soil sampling; and GWC, VWC, and mean daily temperature of soils during the ^13^C-CO_2_ pulse labelling experiment. Submitted as a separate attachment (.xlsx).

**Dataset S2:** Seasonal measurements of tree height and stem diameter increment, stomatal conductance (gs) and light-saturated photosynthesis (A_net_), predawn leaf water potential, (Ψ) needle area, fine root biomass and morphology at the time of ^13^C-CO_2_ pulse labelling. Submitted as a separate attachment (.xlsx).

**Dataset S3:** Carbon isotopic composition of tree needles, fine roots, soil pore CO_2,_ and phospholipid fatty acids (PLFA); total C concentration in needles and fine roots; soil pore CO_2_ concentrations and data used for PLFA quantification during the ^13^C-CO_2_ pulse labelling experiment. Submitted as a separate attachment (.xlsx).

**Fig S1:** Pictures of the mesocosms taken at the end of the first growing season of the trees. Control (green), intermediate water limitation (dark yellow), severe water limitation (brown).

**
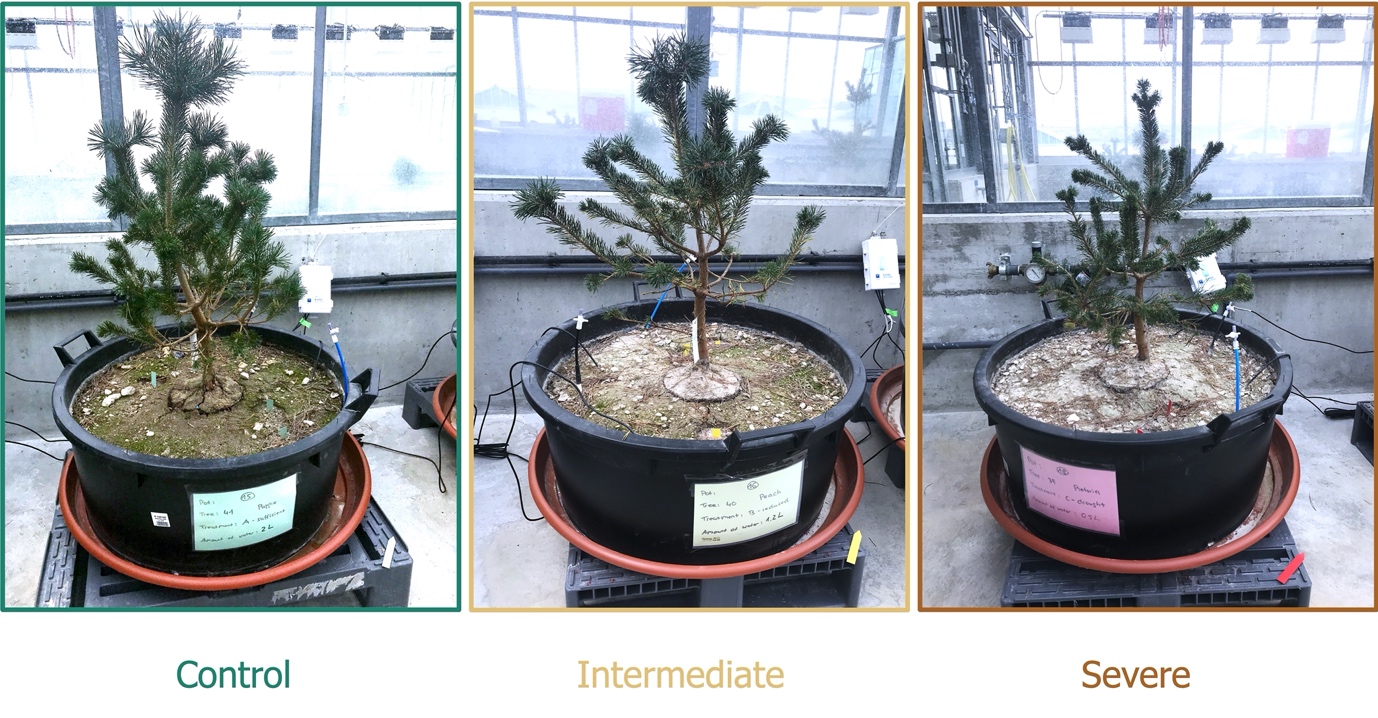
**

**Figure S2:** Picture of Scots pine-forest soil mesocosms during the ^13^C-CO_2_ pulse labelling.

**
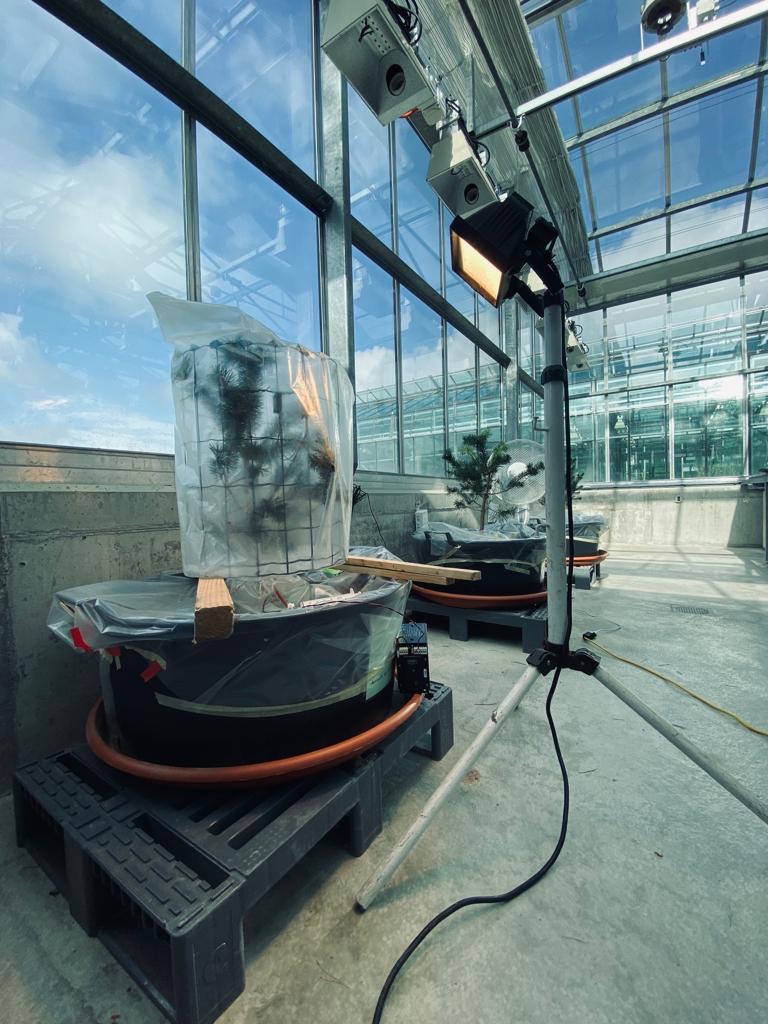
**

**Figure S3:** Visualization of the relationships between a) the volumetric water content and gravimetric water content of all soil samples collected on a seasonal basis, and b) the soil-extractable organic carbon (EOC) and the gravimetric water content of all soil samples collected on a seasonal basis. The different symbols represent the seasonal time points.

**Table S1:** Seasonal greenhouse (GH) air temperature and humidity during the course of the mesocosm experiment. Means as well as minimum and maximum temperatures (min - max) are presented.

| Parameter | **Winter 20** | **Spring 20** | **Summer 20** | **Autumn 20** | **Winter 21** |
| --- | --- | --- | --- | --- | --- |
| GH temperature [°C] | **10.3** (6.4 – 18.7) | **16.5** (8.6 – 26.4) | **21.5** (15.5 – 26.5) | **15.9** (8.9 – 26.5) | **10.8** (7.6 – 23.0) |
| GH humidity [%] | **53** (16 – 91) | **47** (7 – 85) | **62** (46 – 78) | **66** (57 – 94) | **51** (8 – 85) |

**Methods S1: Details on the soil and trees used in the mesocosm experiment (continuation of method description)**

The soil and the stones were collected in November 2018 using an excavator. The upper 3-6 cm deep organic horizon (Oe horizon) was removed, and approximately 4 t of mineral soil was excavated from the upper ~35 cm of the mineral soil. The mineral soil (sand/silt/clay %: 49/43/8; skeletal material: 20-50 %) was transported in bulk bags to the Research Station for Plant Sciences (ETH Zurich, Lindau, Switzerland) and intermediately stored outside on wooden pallets covered for protection against rain. A steel riffle splitter was used to homogenize the soil and remove large stones.

Before being transplanted to the mesocosms, the Scots pine saplings had been growing at the tree nursery of the Swiss Federal Research Institute for Forest, Snow and Landscape Research (WSL, Birmensdorf, Switzerland). The trees were initially grown from seeds in a common potting substrate for two years. In February 2019, the trees were moved to small pots (6 L volume) containing the homogenized mineral soil. The intermediate planting in 6 L pots allowed the trees to acclimate to the new soil substrate during one growing season.

**Methods S2: Automated soil moisture and temperature sensors and soil pore gas sampling system (continuation of method description)**

Each mesocosm was equipped with soil sensors installed at 15 cm depth to measure the volumetric water content and soil temperature at a temporal resolution of 60 minutes (Teros 11, Meter Group, Pullman, WA, USA). The soil sensors were connected to Em50 loggers (Decagon Devices, Pullman, WA, USA). Moreover, a 68 cm hydrophobic capillary membrane segment was placed in the soil at 15 cm depth with a connection to an outlet port to sample soil pore gas (Longepierre et al. 2021; Verhoeven et al. 2018).

**Methods S3:** **Gravimetric water content and concentrations of K_2_SO_4_ extractable soil organic carbon (continuation of method description)**

Gravimetric water content was assessed by weighing a sieved subsample of 10 g of fresh soil before and after drying at 105 °C for 72h. K_2_SO_4_ extractable soil organic carbon (EOC) was extracted from a sieved subsample of 12.5 g of fresh soil with 50 ml of 0.5 M K_2_SO_4_ on a horizontal shaker for 60 min at 180 rpm. The extract was separated from the coarse particles using 150 nm ashless filter paper (Whatman No. 42) and subsequently frozen and stored at -20°C until analysis. For the analysis, the extract was diluted with Milli-Q water (1:5 v/v) and acidified using 2M HCl to enable the removal of carbonates. Soil EOC concentrations were determined via high-temperature combustion on a TOC-L total carbon analyzer (Shimadzu, Kyoto, Japan)*.*

**Methods S4: Aboveground tree growth parameters (continuation of method description)**

The height of the trees was measured from the ground to the shoot apex. The leader shoot was measured from the base of the shoot to the whorl of buds developing at its apex. The stem diameter was measured with a digital caliper seven cm above the ground at two angles, and the mean was taken.

**Methods S5: ^13^C isotopic composition of CO_2_ in gaseous samples (continuation of method description)**

The δ^13^C values of CO_2_ in gaseous samples were measured with a modified Gasbench II periphery (Finnigan MAT, Bremen, D) coupled to an isotope ratio mass spectrometer (Delta^plus^XP; Finnigan MAT); modification as described by Zeeman et al. (2008). Isotope ratio mass spectrometry (IRMS), post-run offline calculation, and drift correction for assigning the final *δ*^13^C values on the Vienna Pee Dee Belemnite (VPDB) scale were done following the “IT principle” as described by Werner and Brand (2001). The *δ*^13^C values of the laboratory air standards were determined at the Max-Planck Institute for Biogeochemistry (Jena, Germany), according to Werner et al. (2001). The linking of the measured *δ*^13^C (and *δ*^18^O) values of CO_2_ gas isolated from air samples relative to the carbonate VPDB scale was done via the Jena Reference Air Standard (JRAS).

**Methods S6: Extraction of Phospholipid Fatty Acids** **(continuation of methods description)**

Extraction of soil microbial phospholipid fatty acids (PLFAs) was performed on the previously frozen soil subsamples. The following samples were selected for extraction: 1 day before the pulse-labelling, 1, 2, 3, and 7 days after. Total PLFAs were extracted from a wet soil subsample of 1.5 g dry weight. ﻿Initial extraction was performed with a mixture (1:2:0.8 v/v/v) ﻿of chloroform (CHCl_3_), methanol (MeOH), and citrate buffer (K_3_Cit / H_3_Cit), whereby samples were left overnight after mixing for phase separation. To separate the lipid fraction from other organics, a second extraction was performed on the extraction residues of the first step by adding chloroform-methanol (CHCl_3_:MeOH, 1:2 v/v) and 1M citrate buffer. The aqueous and organic phases were separated by adding 1.5 mL chloroform and 1.5 mL citrate buffer, mixing, and centrifuging. The organic phase was transferred to clean vials, and subsequent evaporation of solvents was conducted by placing the sample for 30 min on a heating block (40 °C) under a continuous flow of N_2_. Separation of ﻿the lower lipophilic phase into neutral lipids, glycolipids, and phospholipids was achieved by solid phase extraction using silicic acid columns (ISOLUTE^®^ SPE Column, Biotage, Sweden), impregnated with chloroform, and a Supelco Visiprep^TM^ System (Sigma-Aldrich, USA). 500 μl chloroform was added to each sample and the solution was transferred to the columns. 3 x 2.5 ml chloroform was added to separate neutral lipids, followed by 8 x 2.5 ml acetone (AcOH) for glycolipids and 2 x 2.5 ml methanol for phospholipids. Samples were dried for 1h on a heating block (40 °C) under a continuous flow of N_2._ C19:0 (1,2-Dinonadecanoyl-sn-glycero-3-phosphocholine, Sigma-Aldrich, USA) dissolved in methanol-toluene (1:1 v/v) was added (1 mL of 1 μg μL^−1^) as an internal standard for quantification. PLFAs were methylated by adding 1 mL 0.2 M methanolic potassium hydroxide solution (KOH in MeOH) and incubation at 40 °C for 30 min. After cooling to room temperature, neutralization and separation of aqueous and organic phases were conducted. 2 mL n-hexane-chloroform (Hex:CHCl3, 4:1 v/v), 200 μL 1M acetic acid (HAc), and 2 mL ultra-purified water were added. After centrifugation, the upper organic phase was transferred to clean vials. 2 mL n-hexane-chloroform (4:1 v/v) was added two subsequent times for complete sample transfer. Samples were dried for 1h at 40 °C under continuous N_2_ flow. 200 μl isooctane was used to transfer samples to gas chromatography (GC) vials with conical glass inserts. The samples were stored at −20 °C until further analysis.

**Methods S7: ^13^C measurements of Phospholipid Fatty Acids** **(continuation of methods description)**

The *δ*^13^C values of individual PLFA were determined using IRMS. The Trace 1300 GC was connected via a ConFlo IV continuous flow interface (Thermo Fisher Scientific, USA) to a Delta Plus Advantage IRMS (Thermo Fisher Scientific, USA). The GC was equipped with an SPB^TM^ (24028-U, Supelco, Sigma-Aldrich USA) capillary column, 30m x 0.25mm, and 0.25 μm film, and helium was used as a trace gas with 20cm s^-1^.

PLFA *δ*^13^C data were calibrated against internal reference standards. These standards were analyzed in various concentrations at the beginning of every GC sequence and were used together with the internal standard 19:0 for quantifying PLFAs. For the analysis of *δ*^13^C values, we only used PLFAs, which were present in concentrations large enough to ensure reproducibility of *δ*^13^C values in GC-IRMS measurements.

**Methods S8: Total weight of tree biomass compartments (continuation of methods description)**

The total needle biomass at the time of ^13^C-CO_2_ pulse labelling was obtained for each tree based on oven-dried needle weight and the estimation of the total number of needles on the branches of the trees. The total biomass of the stems and branches of each of the trees was estimated based on the oven-dried weight of these components after the final harvest of the mesocosm experiment (in August 2022), by considering the average length of the branches and stem diameter at the time of pulse labelling relative to those measured during the final harvest. The total biomass of living coarse and fine roots in the upper 0-20 cm of the soil in the mesocosms was estimated by two larger soil samples collected after the end of the pulse labelling experiment with a slide-hammer corer (5.5 cm diameter). Before being dried at 70°C and weighed, dead roots were removed, and living roots with a diameter < 2 mm were sorted and scanned using a flatbed scanner (EPSON Expression 11000XL, EPSON, Suwa, Nagano, Japan). The scanned images were then analyzed using the WinRHIZO program (version 2013, Regent Instruments Inc., Chemin Sainte-Foy, Quebec, Canada) to obtain data regarding the length per soil volume, the average diameter of the fine roots (Brunner et al. 2019). The proportion of fine roots recovered in different diameter sizes was analysed as in Meller et al. (2020).

**Methods S9: Soil CO_2_ efflux** **(continuation of methods description)**

The soil CO_2_ efflux was modelled using CO_2_ concentrations, soil moisture, and temperature data as well as Fick’s Law of Diffusion following Van de Broek et al. (2020) and Hicks Pries et al. (2017), (Eq. S3):

$F= - D_{{s; a, i}}\frac{\left( CO_{2} \right)_{a}-\left( CO_{2} \right)i}{\Delta_{z}}$ Eq. S3

where *F* is the CO_2_ flux transported through the surface of the pot (μmol m^-2^ s^-1^), $D_{{s; i, a}}$ is the harmonic average of the effective diffusivity coefficient (D_s_) between a horizontal plane in the soil of each pot (*i*) and the atmosphere (*a*), and $\Delta_{z}$ is the depth of the pot. The effective diffusivity coefficient is calculated considering the following empirical relationship (Eq. S4):

$D_{s}=D_{0}*$ Eq. S4

where D_0_ is the gas diffusion coefficient of CO_2_ in free air at sampling time (m^-2^ s^-^) and is the dimensionless tortuosity factor. We estimated tortuosity as in Moyes and Bowling (2013), = 0.95ε^1.93^, where ε is the air-filled porosity of the soil at sampling time (m^-3^/ m^-3^), and $\Phi_{i}$ is the total soil porosity. The total soil porosity was calculated as $\Phi_{i}= -\rho_{i}/\rho_{p}$, where $\rho_{i}$ is the bulk density of the soil (tm^-3^), and $\rho_{p}$ is the particle density (2.65 tm^-3^). The air-filled porosity was calculated as the difference between the total porosity (m^-3^/ m^-3^) and the water-filled pore space in the pot at sampling time (m^-3^/ m^-3^). For each mesocosm, the gas diffusion coefficient in free air was corrected for variations in temperature and soil moisture (Massman 1998; Van de Broek et al. 2020) with the following equation (Eq. S5):

$D_{0 =}D_{0,stp}\frac{p_{0}}{p} \left( \frac{T}{T_{0}} \right)^{\alpha}$ Eq. S5

where D_0,stp_ is the gas diffusion coefficient for CO_2_ in free air under standard temperature (0°C) and pressure (1 atm) (1.385 x 10^-5^ m^2^ s^-1^), and $\alpha$ represents a coefficient (1.81) (Massman 1998). A constant atmospheric pressure *p* of 1 atm was presumed throughout the experiment and soil temperature *T* was measured continuously.

**References:**

Brunner I, Herzog C, Galiano L, Gessler A (2019) Plasticity of Fine-Root Traits Under Long-Term Irrigation of a Water-Limited Scots Pine Forest. Frontiers in Plant Science 10. doi: 10.3389/fpls.2019.00701.

Hicks Pries CE, Castanha C, Porras RC, Torn MS (2017) The whole-soil carbon flux in response to warming. Science 355: 1420-1423. doi: 10.1126/science.aal1319.

Longepierre M, Feola Conz R, Barthel M, Bru D, Philippot L, Six J, Hartmann M (2021) Mixed Effects of Soil Compaction on the Nitrogen Cycle Under Pea and Wheat. Front Microbiol 12: 822487. doi: 10.3389/fmicb.2021.822487.

Massman WJ (1998) A review of the molecular diffusivities of H_2_O, CO_2_, CH_4_, CO, O_3_, SO_2_, NH_3_, N_2_O, NO, and NO_2_ in air, O_2_ and N_2_ near STP. Atmospheric Environment 32: 1111-1127. doi: <https://doi.org/10.1016/S1352-2310(97)00391-9>.

Meller S, Frossard E, Spohn M, Luster J (2020) Plant nutritional status explains the modifying effect of provenance on the response of beech sapling root traits to differences in soil nutrient supply. Frontiers in Forests and Global Change 3: 110.

Moyes AB, Bowling DR (2013) Interannual variation in seasonal drivers of soil respiration in a semi-arid Rocky Mountain meadow. Biogeochemistry 113: 683-697. doi: 10.1007/s10533-012-9797-x.

Van de Broek M, Ghiasi S, Decock C, Hund A, Abiven S, Friedli C, Werner RA, Six J (2020) The soil organic carbon stabilization potential of old and new wheat cultivars: a ^13^CO2-labeling study. Biogeosciences 17: 2971-2986. doi: 10.5194/bg-17-2971-2020.

Verhoeven E, Decock C, Barthel M, Bertora C, Sacco D, Romani M, Sleutel S, Six J (2018) Nitrification and coupled nitrification-denitrification at shallow depths are responsible for early season N2O emissions under alternate wetting and drying management in an Italian rice paddy system. Soil Biology and Biochemistry 120: 58-69.

Werner RA, Brand WA (2001) Referencing strategies and techniques in stable isotope ratio analysis. Rapid Communications in Mass Spectrometry 15: 501-519.

Zeeman MJ, Werner RA, Eugster W, Siegwolf RTW, Wehrle G, Mohn J, Buchmann N (2008) Optimization of automated gas sample collection and isotope ratio mass spectrometric analysis of δ^13^C of CO_2_ in air. Rapid Communications in Mass Spectrometry 22: 3883-3892. doi: <https://doi.org/10.1002/rcm.3772>.
